# Higher emotional granularity relates to greater inferior frontal cortex cortical thickness in healthy, older adults

**DOI:** 10.3758/s13415-023-01119-y

**Published:** 2023-07-13

**Authors:** Sladjana Lukic, Eena L. Kosik, Ashlin R. K. Roy, Nathaniel Morris, Isabel J. Sible, Samir Datta, Tiffany Chow, Christina R. Veziris, Sarah R. Holley, Joel H. Kramer, Bruce L. Miller, Dacher Keltner, Maria Luisa Gorno-Tempini, Virginia E. Sturm

**Affiliations:** 1 Memory and Aging Center, Department of Neurology, University of California, San Francisco, CA USA; 2 Adelphi University, Hy Weinberg Center, Suite 136, Garden City, NY 11530-0701 USA; 3 Psychology Department, San Francisco State University, San Francisco, CA USA; 4 Department of Psychology, University of California, Berkeley, CA USA

**Keywords:** Emotion granularity, Affect labeling, Orbitofrontal cortex, Insula, Well-being, Aging

## Abstract

Individuals with high emotional granularity make fine-grained distinctions between their emotional experiences. To have greater emotional granularity, one must acquire rich conceptual knowledge of emotions and use this knowledge in a controlled and nuanced way. In the brain, the neural correlates of emotional granularity are not well understood. While the anterior temporal lobes, angular gyri, and connected systems represent conceptual knowledge of emotions, inhibitory networks with hubs in the inferior frontal cortex (i.e., posterior inferior frontal gyrus, lateral orbitofrontal cortex, and dorsal anterior insula) guide the selection of this knowledge during emotions. We investigated the structural neuroanatomical correlates of emotional granularity in 58 healthy, older adults (ages 62–84 years), who have had a lifetime to accrue and deploy their conceptual knowledge of emotions. Participants reported on their daily experience of 13 emotions for 8 weeks and underwent structural magnetic resonance imaging. We computed intraclass correlation coefficients across daily emotional experience surveys (45 surveys on average per participant) to quantify each participant’s overall emotional granularity. Surface-based morphometry analyses revealed higher overall emotional granularity related to greater cortical thickness in inferior frontal cortex (*p*_FWE_ < 0.05) in bilateral clusters in the lateral orbitofrontal cortex and extending into the left dorsal anterior insula. Overall emotional granularity was not associated with cortical thickness in the anterior temporal lobes or angular gyri. These findings suggest individual differences in emotional granularity relate to variability in the structural neuroanatomy of the inferior frontal cortex, an area that supports the controlled selection of conceptual knowledge during emotional experiences.

**Supplementary information:**

The online version contains supplementary material available at 10.3758/s13415-023-01119-y.

## Introduction

Emotions are multisystem responses (Levenson, 1999) that people can understand and experience in various ways. Individuals differ in the richness of their emotional experiences (emotional awareness), the variety of feelings they have over time (i.e., emodiversity), and the clarity (emotional clarity) or opacity (emotional complexity) with which they feel their emotions (Grossmann et al., 2019; Grühn et al., 2013; Hoemann et al., 2021c; Ong et al., 2018; O’Toole et al., 2020; Quoidbach et al., 2014). How people describe their feelings is complex but likely reflects their underlying mental representations of emotions (Hoemann et al., 2021c). Emotional granularity refers to the degree to which people experience emotions with precision by activating underlying conceptual (or semantic) knowledge (Barrett et al., 2001; Tugade e al., 2004). Because individuals with higher emotional granularity have more refined conceptual knowledge of emotional experience, they are better able to differentiate among the myriad feelings that arise in everyday life.

Conceptual knowledge is comprised of verbal and nonverbal information, but there are ongoing debates regarding the extent to which conceptual knowledge can ever be separated from language (Jackendoff, 2010, Levelt, 1999; Lukic & Borghensani, 2023). People must use words to describe their inner states, and language is a window into how they understand their emotional experiences using conceptual knowledge (Barrett, 2004). While some individuals express their feelings with broad linguistic strokes and do not differentiate among various emotions, others make fine-grained verbal distinctions among even similar types of emotional experiences (Barrett et al., 2001). The words we use to make sense of and articulate our emotional experiences shape our lives in powerful ways. People with higher emotional granularity report a greater diversity of experiences in everyday life (Hoemann et al., 2023) and enjoy numerous mental and physical health benefits (Barrett et al., 2001; Demiralp et al., 2012; Feldman, 1995; Hoemann et al., 2021a, b; Kashdan et al., 2015; Kimhy et al., 2014; Oh & Tong, 2020; Pond et al., 2012; Suvak et al., 2011; Tan et al., 2022; Tomko et al., 2015; Tugade et al., 2004). Although relatively stable across adulthood (Grühn et al., 2013; Hay & Diehl, 2011; Willroth et al., 2020), emotional granularity may continue to develop over the life course (Mankus et al., 2016; Nook et al., 2017, 2018; Ready et al., 2008).

Normal variation in brain structure and function may underlie individual differences in emotional granularity. To have high emotional granularity, one must not only acquire rich conceptual knowledge of emotions but also use this knowledge with skill (Barrett, 2004; Lindquist & Barrett, 2008; Lee et al., 2017). Whereas the anterior temporal lobes and angular gyri play central roles in the representation of conceptual knowledge of emotions, people, and objects (Binder & Desai, 2011; Gorno-Tempini et al., 2004; Guo et al., 2013; Kumfor et al., 2013; Olson et al., 2007; Patterson et al., 2007; Rice et al., 2015; Younes et al., 2022), the inferior frontal cortex (IFC) is essential for the controlled selection of information among competing alternatives stored in semantic knowledge (Lau et al., 2008; Thompson-Schill et al., 1997). The IFC is a lateral prefrontal region that includes areas within the posterior inferior frontal gyrus (pars opercularis and pars triangularis or Areas 44/45), lateral orbitofrontal cortex (pars orbitalis and lateral orbitofrontal areas or Areas 47/12, regions often referred to together as “ventrolateral prefrontal cortex”), and dorsal anterior insula (Aron et al., 2004; Levy & Wagner, 2011). A key hub in a frontoparietal “stop” network, the IFC supports behavioral inhibition, cognitive control, and emotion regulation (Aron et al., 2014; Dörfel et al., 2014; Li et al., 2021; McRae et al., 2011; Ochsner et al., 2004; Phan et al., 2005; Picó-Pérez et al., 2019; Frank et al., 2014; Hooker & Knight, 2006). The IFC also activates when people use language to label and make meaning of the emotions of others (Brooks et al., 2017; Goldin et al., 2008; Hariri et al., 2000; Lieberman et al., 2005, 2007; Phan et al., 2005; Torre & Lieberman, 2018). Translating our own feelings, or the feelings of others, into words requires us to select relevant emotion concepts. The IFC may be crucial for sorting through semantically similar alternatives and discarding those that do not best capture the feelings at hand.

In the present study, we examined whether emotional granularity is related to the structural anatomy of IFC and connected regions. Although there are no previous, structural, neuroimaging studies of emotional granularity, one previous study found that healthy, young adults with higher emotional awareness had greater cortical thickness in the orbitofrontal cortex, anterior temporal lobes, and ventromedial prefrontal cortex (Smith et al., 2018), evidence that individual differences in brain structures relate to emotion reporting tendencies (Lee et al., 2017). Electroencephalography studies also have found that individual differences in emotional granularity relate to distinct spectral patterns in the frontal lobes during affective processing (Lee et al., 2017; Wang et al., 2020). Because measures of emotional granularity require people to use discretion in their endorsement—and denial—of specific types of emotional experience, we hypothesized that participants with higher emotional granularity would have greater cortical thickness in IFC, a region that may support the controlled selection of emotion concepts during experience reporting (Thompson-Schill et al., 1997). The IFC has connections with both the anterior temporal lobes and angular gyri (Aron et al., 2004; Duvernoy, 1999; Du et al., 2020; Leng et al., 2016; Mesulam, 2000; Petrides & Pandya, 2002; Von Der Heide et al., 2013), and we included these as control regions. Whereas both areas are critical for conceptual knowledge of emotions (Binder & Desai, 2011), the angular gyri also are important for emotion regulation and nonverbal behavioral control (Barrett, 2017; Kohn et al., 2014; Li et al., 2021), making both regions stringent comparisons in our investigation of the neural correlates of emotional granularity.

## Methods

### Participants

Fifty-eight, healthy, older adults (aged 62–84 years) participated in the present study. Participants were volunteers in the Hillblom Healthy Aging Network, a longitudinal study of healthy aging at the University of California, San Francisco (UCSF). All participants were recruited from the community and underwent an extensive, multidisciplinary, team evaluation that included a clinical history, neurological examination, neuropsychological testing, informant-based functional interview, and neuroimaging. Participants had no history of speech-language, learning, neurological, or psychiatric disorders. On the Clinical Dementia Rating scale (CDR)—an informant-based interview that assesses daily functioning (Morris, 1997)—all participants had a CDR total score of zero, which indicates intact daily functioning (e.g., paying bills, personal care, cooking meals, and scheduling appointments)—thus, no evidence of decline that might suggest an incipient neurodegenerative disease. Participants also completed the Mini-Mental State Examination (MMSE), a brief screen of cognitive functioning (Folstein et al., 1975), and all scored 25 (out of 30) or higher (Table 1). The study procedures were approved by the UCSF Committee on Human Research, and all participants provided written, informed consent. Participants were financially compensated for their participation.

**Table 1:** Demographic information and cognitive scores for the participants. Means (*M*) and standard deviations (*SD*) are provided

	*M* (*SD*)
*N*	58
Age (yr)	74.7 (4.3)
Sex (female / male)	38 / 20
Handedness (right / left)	54 / 4
Education (yr)	17.4 (1.9)
Clinical dementia rating scale: total score	0. 0 (0.0)
Mini-Mental State Examination (/30)	29.2 (1.1)
California Verbal Learning Test-II: Delayed Recall (/16)	11.5 (3.5)
Benson figure copy 10-minute Recall (/17)	11.4 (2.3)
Benson figure copy (/17)	15.5 (0.7)
Modified trails (# of correct lines per minute)	40.4 (15.1)
Modified trails errors	0.2 (0.6)
Phonemic fluency (# correct in 60 seconds)	16 (4)
Semantic fluency (# correct in 60 seconds)	22 (4)
Design fluency correct (# correct in 60 seconds)	12.2 (3.7)
Digits backward	5 (1.4)
Boston Naming Test Spontaneous Correct (/15)	15 (0.7)

### Procedure

The data were collected as a part of an 8-week positive emotion intervention study (Sturm et al., 2020). In that study, participants completed daily experience surveys and were randomly assigned to one of two groups. In the control group, participants took a weekly outdoor walk. In the intervention group, participants also took a weekly outdoor walk but were instructed to orient their walks to increase experiences of awe. Participants also completed preintervention baseline measures, which were not used in the present study.

### Measures

#### Overall emotional granularity

On each afternoon of the study, participants received an email with a link to an online survey regarding their daily emotional experiences. They were asked to rate their experience of a wide range of emotions that day on a 7-point Likert scale (1 = *not at all*; 4 = *a little*; 7 = *a lot*). They reported on their daily experience of 13 emotions, which included eight positive emotions (awe, amusement, compassion, pride, calm, contentment, joy, and gratitude) and five negative emotions (anger, annoyance, anxiety, fear, and sadness). These emotions were selected to include a wide range of positive and negative emotions that spanned the dimensions of arousal from calm to activated.

**Fig. 1:**
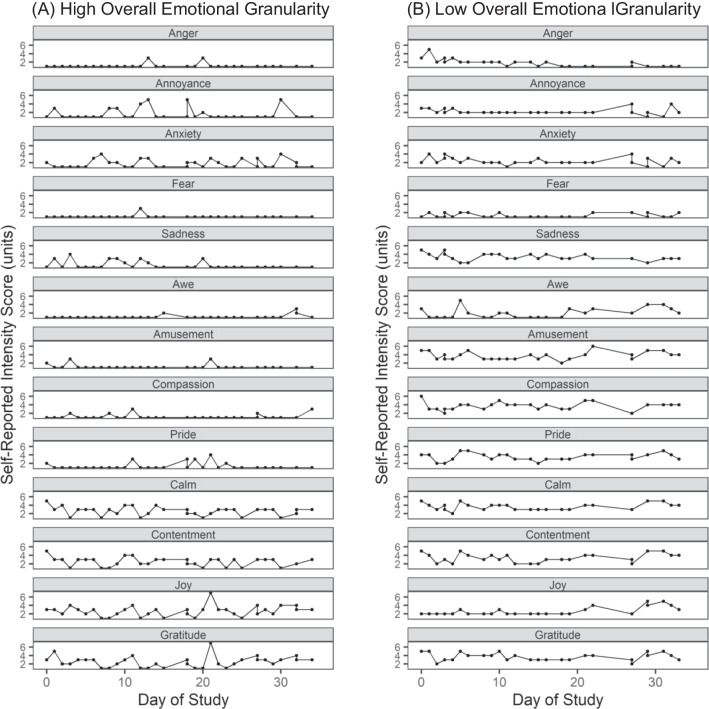
Emotional experience surveys for two example participants. Daily survey ratings for two participants are provided as examples to illustrate how individual differences in day-to-day positive and negative emotional experiences contribute to overall emotional granularity scores. **A** Participant with high overall emotional granularity reported highly differentiated, nonoverlapping emotional experiences. In this participant, endorsement of one emotion was less likely to be accompanied by endorsement of other emotions on that day. **B** Participant with low overall emotional granularity reported less differentiated emotional experiences. In this participant, endorsement of one emotion was often accompanied by endorsement of other emotions on that day

Consistent with previous studies (Demiralp et al., 2012; Hoemann et al., 2021a, 2023; Lee et al., 2017; Nook et al., 2018; Tugade et al., 2004), we calculated a measure of overall emotional granularity by quantifying the degree to which participants distinguished among different types of emotional experience, as measured by the daily surveys. Our primary measure was an intraclass correlation coefficient (ICC) that was computed across the positive and negative emotion words to create a measure of overall emotional granularity. Because previous studies have found that older adults may experience more mixed emotions and less opposition between emotion states of different valences (Carstensen et al., 2000), we calculated overall emotional granularity not only by quantifying how the participants made separate distinctions among the positive words and the negative words but by also including measures of the degree to which they differentiated between positive and negative emotional experiences. The ICCs were Fisher *r*-to-*z* transformed to fit the variable to a normal probability distribution. These transformed values were subtracted from 1 such that lower values reflected lower granularity and higher values reflected higher granularity. All ICC scores were between 0 and 1. This was the primary measure of emotional granularity that we used in our analyses (Fig. 1).

#### Average emotional granularity

Because there is no consensus about whether overall emotional granularity should be computed across all emotion terms (Kimhy et al., 2014) or first across same-valence terms and then averaged (Lee et al., 2017), we also computed measures of positive emotional granularity (i.e., positive-positive words) and negative emotional granularity (i.e., negative-negative words) and then averaged these two scores. We used this to confirm our cortical thickness results held using an alternate measure of emotional granularity.

#### Overall emotional experience intensity

For each participant, we computed an overall emotional experience intensity score by averaging the intensity ratings for all emotions across the entire sampling period. Higher scores indicated more intense daily emotional experiences (maximum score = 7). We used this as a covariate in our analyses of emotional granularity.

### Neuroimaging acquisition and preprocessing

Participants underwent research-quality structural magnetic resonance imaging as a standard part of their annual research visit in the longitudinal healthy aging study. The scans were obtained within 18 months of their enrollment in the intervention study. Neuroimaging was performed using a Siemens Trio 3-Tesla scanner equipped with a 12-channel head coil at the UCSF Neuroscience Imaging Center. A T1-weighted MP-RAGE structural scan was acquired with an acquisition time = 8 minutes and 53 seconds, sagittal orientation, a field of view of 160 x 240 x 256 mm with an isotropic voxel resolution of 1 mm^3^, repetition time = 2,300 ms, echo time = 2.98 ms, inversion time = 900 ms, flip angle = 9 °.

After a visual inspection, no participants were excluded for extensive white matter disease or significant motion artifacts. Neuroimaging data preprocessing and statistical analyses were performed using the Computational Anatomy Toolbox (CAT12; http://dbm.neuro.uni-jena.de/cat) within Statistical Parametric Mapping software (SPM12; http://www.fil.ion.ucl.ac.uk/spm/software/spm12). The T1-weighted images were bias-field corrected, skull-stripped, aligned to the Montreal Neurological Institute (MNI) standard space, and segmented into gray matter, white matter, and cerebrospinal fluid. We used the projection-based thickness method, which accounts for partial volume information, sulcal blurring, and sulcal asymmetry (Dahnke et al., 2013), to compute cortical thickness—a measure sensitive to brain tissue integrity in healthy aging (Hutton et al., 2009). The local maps were resampled and smoothed by using a 15-mm Gaussian heat kernel (Yotter et al., 2011).

### Statistical analyses

#### Association between overall emotional granularity and cortical thickness

To test our hypotheses, we selected regions of interest (ROIs) from the Brainnetome atlas (https://atlas.brainnetome.org/bnatlas.html) that capture all relevant areas of the IFC: (1) posterior inferior frontal gyrus, which included pars opercularis and pars triangularis (Areas 44/45); (2) lateral orbitofrontal cortex, which included pars orbitalis (Area 47) and Area 12; (3) lateral orbitofrontal cortex, which included Area 11; and (4) dorsal anterior insula (Aron et al., 2004; Barrett, 2017; Picó-Pérez et al., 2019). We also conducted two control analyses that focused on the anterior temporal lobes and angular gyri, regions associated with conceptual knowledge of emotions and emotion regulation (Barrett, 2017; Binder & Desai, 2011).

We conducted surface-based morphometry analyses in CAT12 to examine whether there was a positive association between overall emotional granularity and cortical thickness. We ran linear-regression analyses to test whether overall emotional granularity predicted vertex-wise cortical thickness in each ROI (https://neuro-jena.github.io/cat12-help/). Nuisance covariates included age, sex, education, group (control or intervention), time interval (the number of days between the first survey date and the MRI), and the overall emotional experience intensity score. Consistent with previous studies, we included the overall emotional experience intensity score as a covariate to ensure that we were examining the extent to which participants differentiated among different emotional states rather than the intensity with which they experienced them (Demiralp et al., 2012; Kimhy et al., 2014). Significance was set to peak-level and cluster-level family-wise error (FWE) correction at *p* < 0.05. The anatomical locations of significant clusters were confirmed by using the Desikan-Killiany DK40 Atlas, a surface-based atlas (https://neuro-jena.github.io/cat12-help/). To confirm that our results held with average emotional granularity, an alternate form of emotional granularity, we next repeated these analyses in ROIs where we found significant associations with overall emotional granularity (including the same nuisance covariates as in our original analyses).

Although ROI-based analyses improved our power by reducing the number of multiple comparison corrections (Saxe et al., 2006), we also conducted a whole-brain exploratory analysis to confirm that no other regions were associated with overall emotional granularity. By first examining *a priori* ROIs and then expanding our search to the whole brain, we also reduced the likelihood of inflated correlations and spurious brain-behavior correlations, which have been a point of contention in neuroimaging studies (Marek et al., 2022; Vul et al., 2009). For this analysis, we relaxed the peak-level significance threshold to *p* < 0.005 (uncorrected) and set a cluster-level extent threshold of >45 voxels. To confirm our results had a positive linear association with overall emotional granularity, we extracted the mean cortical thickness in significant clusters and plotted them against overall emotional granularity.

#### Follow-up analyses

Given that the emotional experience data were collected as a part of a positive emotion intervention study, in addition to including group as a covariate in our analyses, we also ran tests to confirm that random assignment to the intervention or control group did not influence any of our results (controlling for age, sex, education, and overall emotional experience intensity).

## Results

Each participant completed multiple daily emotional experience surveys (*M* = 45, *SD* = 13, range = 9–59) over the course of the 8-week study. Overall emotional granularity scores varied across the sample (*M* = 0.90, *SD* = 0.07, range = 0.64–1.00). Participants overall emotional experience intensity scores suggested they experienced moderate levels of emotional experience on average across the sampling period (*M* = 3.23, *SD* = 0.83, range = 1.42–4.77). In keeping with previous studies of healthy aging, participants reported higher levels of positive emotional experience (*M* = 4.06, *SD* = 1.18) than negative emotional experience (*M* = 1.89, *SD* = 0.74) on a day-to-day basis (Carstensen et al., 2000; Mroczek & Kolarz, 1998). Overall emotional granularity was not correlated with overall emotional experience intensity, *r* = 0.10, *p* = 0.44.

### Higher overall emotional granularity related to greater cortical thickness in IFC

**Fig. 2:**
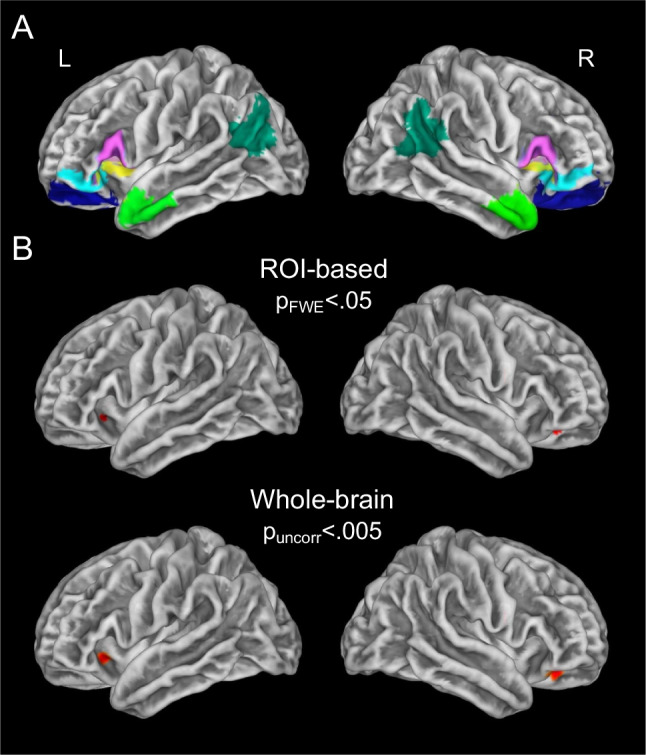
Structural correlates of overall emotional granularity. **A** The neuroimaging analyses of overall emotional granularity focused on the following IFC ROIs: (1) posterior inferior frontal gyrus, which included pars opercularis and pars triangularis (areas 44/45; pink); (2) lateral orbitofrontal cortex, which included pars orbitalis (Area 47) and Area 12 (cyan); (3) lateral orbitofrontal cortex, which included Area 11 (blue); and (4) dorsal anterior insula (yellow). In control analyses, we also examined the anterior temporal lobes (light green) and angular gyri (dark green). **B** Higher overall emotional granularity was associated with greater cortical thickness in the left and right lateral orbitofrontal cortex when controlling for age, sex, education, group (control or intervention), time interval (the number of days between the survey date and the MRI), and overall emotional experience intensity. The left-lateral orbitofrontal cortex cluster extended into the dorsal anterior insula according to more parcellated surface-based atlases. The images were peak-level and cluster-level corrected (*p*_FWE_ < 0.05) for the ROI analysis, and peak-level thresholded at *p* < 0.005, uncorrected, for whole-brain analysis. The color maps (red) reflect the maximum *T* value in each analysis

Higher overall emotional granularity was associated with greater cortical thickness in the left (*T* = 3.25, *k* = 14) and right (*T* = 3.47, *k* = 27) lateral orbitofrontal cortex (*p*_FWE_ < 0.05; Fig. 2; Supplementary Fig. 1). In the left-lateral orbitofrontal cortex, the cluster extended into the dorsal anterior insula according to more parcellated surface-based atlases. Higher overall emotional granularity was not associated with greater cortical thickness in the anterior temporal lobes or angular gyri, however. A follow-up, whole-brain analysis (*p* < 0.005, uncorrected) of overall emotional granularity revealed the same clusters in the left and right lateral orbitofrontal cortex but no additional clusters. A similar pattern of results, although weaker, was found by using the average emotional granularity score (Supplementary Fig. 2).

### Follow-up analyses

To confirm that group assignment did not account for any of our neuroimaging results, we compared the intervention and control groups and found they were similar in their demographic and cognitive profiles (Supplementary Table 1). There were no differences between the intervention and control groups in their mean levels of overall emotional granularity, *b* = 0.49, *t*(53) = 0.53, *p* = 0.601, and they reported similar levels of overall emotional experience intensity (Supplementary Table 2).

## Discussion

The present study uncovered a novel association between emotional granularity and IFC structural neuroanatomy in healthy, older adults. Higher overall emotional granularity related to greater cortical thickness in the right- and left-lateral orbitofrontal cortex, with the cluster in the left hemisphere extending into the dorsal anterior insula. Cortical thickness in the anterior temporal lobes and angular gyri were not associated with overall emotional granularity, and whole-brain analyses revealed no other associated regions. The neural correlates of overall emotional granularity (which was computed with an ICC across all emotions and included positive-positive, negative-negative, and positive-negative word pairs) were consistent with those found when we used an alternate measure of average emotional granularity (which was calculated by averaging the ICCs for the positive-positive and negative-negative word pairs only). Overall emotional granularity had a much stronger correlation with IFC cortical thickness than average emotional granularity, however.

People who are high in emotional granularity must not only have access to a rich semantic library of emotion concepts, but they also must use that knowledge effectively to select the words that capture their inner experiences and to reject those that do not (Lindquist & Barrett, 2008). The anterior temporal lobes and angular gyri are key structures in the representation of multimodal conceptual knowledge (Binder & Desai, 2011; Barrett & Satpute, 2013; Buckner et al., 2008; Li et al., 2014; Raichle et al., 2001; Von Der Heide et al., 2013), but cortical thickness in these regions was not associated with emotional granularity. While the angular gyri also promote emotion regulation by helping the IFC to execute targeted action plans (Kohn et al., 2014), our results did not suggest that these posterior regions play a central role in the verbalization of emotional experience, as captured by our measure of emotional granularity.

Higher overall emotional granularity was instead associated with greater cortical thickness in the IFC, a lateral prefrontal region that plays a central role in the selection of conceptual knowledge (Lau et al., 2008) and participates in a distributed neural network that supports emotion regulation, cognitive control, and behavioral inhibition (Aron et al., 2004, 2014; Levy & Wagner, 2011). As the overall emotional granularity score was calculated from participants’ self-reported ratings of daily emotional experience rather than from free response or measures that captured their full range of conceptual knowledge of emotions, these results suggest the IFC may be important for emotional granularity given its role in the controlled selection of semantic concepts (Badre & Wagner, 2007; Brooks et al., 2017). Within the IFC, areas in bilateral lateral orbitofrontal cortex were associated with overall emotional granularity. Whereas the right lateral orbitofrontal cortex has strong projections to premotor cortex and may play a critical role in nonverbal emotion concepts (Zald et al., 2014), the left-lateral orbitofrontal cortex has robust connections with language centers in the inferior frontal gyrus (e.g., Broca’s area) and anterior temporal lobes (Duvernoy, 1999; Du et al., 2020; Leng et al., 2016; Mesulam, 2000; Petrides & Pandya, 2002) and may play a central role in verbal conceptual knowledge.

In our sample of healthy, older adults, overall emotional granularity had a stronger correlation with IFC cortical thickness than average emotional granularity. Although some studies suggest that mixed emotional experiences increase with age as older adults become more comfortable with blended positive and negative emotional experiences (Schneider & Stone, 2015), others conclude greater precision in parsing emotional experiences confers socioemotional advantages and resiliency (Coifman et al., 2007) and may increase with age (Ready et al., 2008). As the overall granularity score included the covariance of positive and negative words (in addition to within-valence measures of covariance, which were captured with the average emotional granularity score), we speculate that differentiating among all types of emotional experience may be critical for emotional granularity in the later decades of life, when mixed feelings may become more common or pronounced.

Using words to express feelings is an effective way of managing emotions (Kashdan et al., 2015; Pennebaker, 1997; Pennebaker et al., 1997) that engages IFC (Etkin et al., 2006; Hariri et al., 2000; Lieberman et al., 2007; Taylor et al., 2006). While functional neuroimaging studies have detected transient IFC activity during emotion regulation and affect labeling tasks (McRae et al., 2011; Hariri et al., 2000; Ochsner et al., 2004), our results suggest that using words to report emotional experience in a precise and detailed way also relates to more enduring differences in the structural architecture of bilateral IFC. The older participants in our study had a lifetime to accrue and use conceptual knowledge of emotions (Ready et al., 2006) and, potentially, to develop and refine the structural and functional connections that the IFC has with the anterior temporal lobes (Duvernoy, 1999; Du et al., 2020; Leng et al., 2016; Mesulam, 2000; Petrides & Pandya, 2002) and angular gyri (Seghier, 2013; Zald et al., 2014). Although it is likely that greater cortical thickness in IFC could encourage more precise labeling of feeling states, it also is possible that individuals who describe their subjective experiences with more precise language ultimately develop greater cortical thickness in IFC. The present findings cannot elucidate the causal mechanisms driving this association, but our results may offer new inroads into the biological basis of emotional granularity in the later years of life.

Previous studies have found mixed results regarding the socioemotional lives of older adults, but our results suggest emotional granularity relates to the structural integrity of IFC in those on a salutary aging trajectory. For some people, the later years of life may yield social and emotional benefits, but for others, feelings of disconnection and loneliness can permeate daily experience (Cacioppo et al., 2014; Carstensen et al., 2011; Charles et al., 2001; Mather, 2012; Shiota & Levenson, 2009). There may be variability in the affective lives of older adults because of underlying differences in brain structure and function, and neuroimaging studies of socioemotional systems also find mixed results in people of advanced age. Although some studies find smaller gray matter volume in the IFC (Allen et al., 2005; Salat et al., 2004) and anterior temporal lobes (Fjell et al., 2009) in older adults, others suggest relative preservation in these areas (Lemaitre et al., 2012; Pressman et al., 2016; Raz et al., 2004). Functional neuroimaging studies also have come to different conclusions regarding the role of IFC, as well as other regions, in emotions across the lifespan. While some have found that older adults exhibit elevated prefrontal activity during cognitive and affective tasks (Mather & Carstensen, 2005; Nashiro et al., 2012), others have shown lower IFC engagement during emotion regulation paradigms in people of older age (van Reekum et al., 2018).

Heterogeneous aging samples may have contributed to the mixed conclusions in behavioral and neuroimaging studies of socioemotional aging. Neurodegenerative diseases (e.g., Alzheimer’s disease) become increasingly common in the later years of life and can affect brain regions that support emotion regulation and experience (Seeley et al., 2009). Unlike many previous studies, our sample included older adults who had undergone extensive neurological, neuropsychological, and neuroimaging evaluations to ensure that they were free of even subtle cognitive decline. As even preclinical neurodegenerative changes can impact socioemotional functioning (Chow et al., 2023; Fredericks et al., 2018), some of the mixed results in the healthy aging literature may be the unwitting inclusion of some older adults with very early neurodegenerative changes in otherwise healthy aging samples. Our findings suggest that there are associations between the structural brain anatomy and emotional granularity in older adults without cognitive deficits or other clinical markers of functional impairment.

This study has several limitations to consider. First, we investigated the neural correlates of overall emotional granularity but did not include measures of positive or negative emotional granularity. While the merits of positive emotional granularity are still debated—with some arguing that it is less important for well-being (Barrett et al., 2001) and others suggesting that it enhances coping (Tan et al., 2022; Tugade et al. 2004)—negative emotional granularity may be especially critical for mental health (Barrett & Gross, 2001; Barrett et al., 2001; Thompson et al., 2021). In healthy aging, older adults often report lower and less variable negative emotional experiences than younger adults (Charles et al., 2001; Grühn et al., 2013) and increasing attention to positive information with age (Mather & Carstensen, 2005). A more granular experience of positive emotions may actually impede the ability to savor pleasant moments (Starr et al., 2017), but whether higher positive emotional granularity attenuates the positivity effect in the later years of life warrants future investigation.

Second, the emotional experience data that we used were obtained as part of a positive emotion intervention study (Sturm et al., 2020). Because emotional granularity is usually considered trait-like (Barrett, 1998; Feldman, 1995; Tugade et al., 2004), it is unlikely that modifying participants’ emotional experience would alter the process by which they report it using conceptual knowledge. In addition, we controlled for their random group assignment in our analyses and conducted follow-up analyses to confirm that the intervention itself did not influence the correlations between IFC cortical thickness and emotional granularity that we detected. Additional research may be needed, however, to confirm the generalizability of our results in other contexts.

Third, our primary goal was to identify the structural brain correlates of emotional granularity in older adults rather than to examine age-related associations. Our cross-sectional sample of healthy older adults had a relatively narrow age range in which to explore associations between age and emotional granularity, but there are likely differences in how older adults engage the IFC, anterior temporal lobes, and angular gyri during cognitive and affective tasks (Lacombe et al., 2015; Seghier, 2013). While most previous studies of emotional granularity have focused on younger adults (Barrett et al., 2001, 2007; Boden et al., 2013; Kang & Shaver, 2004; Kashdan et al., 2010; Kashdan & Farmer, 2014; Pond et al., 2012), relatively less is known about emotional granularity in the later years of life (Grühn et al., 2013; Mankus et al., 2016; Ong & Bergeman, 2004; Ready et al., 2008; Starr et al., 2017). Knowledge of emotion concepts becomes elaborated across development, with some evidence for nonlinear changes across childhood and adolescence but increasing sophistication in adulthood (Carstensen et al., 2000; Nook et al., 2017, 2018). Some studies have shown that emotional granularity increases with age (Mankus et al., 2016; Ready et al., 2008), but others suggest stability (Grühn et al., 2013; Hay & Diehl, 2011; Willroth et al., 2020). Future studies that examine whether emotional granularity changes over the life course in relation to brain integrity are needed to determine how emotional granularity might differ between healthy and pathological aging.

The present study suggests the way we parse our feelings relates to the brain’s structural anatomy. Emotional experience, like other mental states, is associated with conceptual knowledge that we acquire and refine across the lifespan (Barrett, 2014; Ekman & Cordaro, 2011; Gross, 1998; Keltner & Gross, 1999; Koole & Aldao, 2016; Levenson, 1994; Tracy & Randles, 2011). In our study, participants who used words to label their emotional experiences with greater precision had greater cortical thickness in IFC, which suggests there is a longstanding association between emotional experiences and brain structure. Our results contribute to current models of the neural circuitry of emotional granularity and help to delineate how language and emotion interact in the aging brain.

### Supplementary information

Below is the link to the electronic supplementary material.

Supplementary file1 (DOCX 491 KB)

## Declarations

### Rights and permissions

Data requests can be submitted through the UCSF Memory and Aging Center Resource Request form: http://memory.ucsf.edu/resources/data. Academic, not-for-profit investigators with Institutional Review Board approval from the UCSF Human Research Protection Program (HRPP) can request data for research studies. The UCSF HRPP will not review the application until the UCSF Memory and Aging Center Executive Committee has signed off on the proposal and consent form. Data are not publicly available because they contain information that could compromise the privacy of the participants. The experiment was not preregistered.
