# Supplementary material for: Higher emotional granularity relates to greater inferior frontal cortex cortical thickness in healthy, older adults
Source: Cogn Affect Behav Neurosci. Author manuscript; Available in PMC 2023 Nov 1. (PMC10545583; doi:10.3758/s13415-023-01119-y)
Supplement: Supplementary Material [file NIHMS1930093-supplement-Supplementary_Material.docx]

Supplementary Materials

**Supplementary Figure 1.** *Positive Association Between Lateral Orbitofrontal Cortex Cortical Thickness and Overall Emotional Granularity.* Higher overall emotional granularity correlated with greater cortical thickness in lateral orbitofrontal cortex (OFC). We extracted the mean cortical thickness of the left and right lateral OFC clusters that were associated with overall emotional granularity in the imaging analyses. Scatterplots are provided to illustrate the positive associations that cortical thickness in these areas had with overall emotional granularity (*p*_FWE_*<*.05, corrected).

**
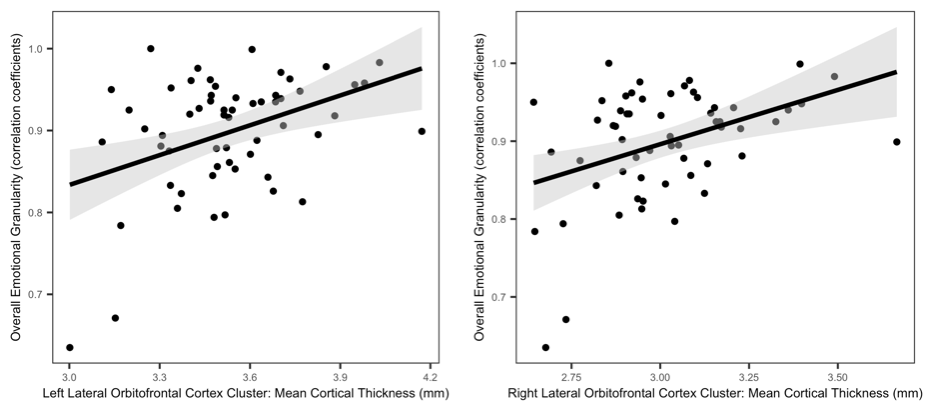
**

**Supplementary Figure 2.** *Structural Correlates of Average Emotional Granularity*. When we instead computed emotional granularity as the average of positive emotional granularity (i.e., positive-positive words) and negative emotional granularity (i.e., negative-negative words), we repeated our analyses in the ROIs that had significant associations with overall emotional granularity in our original analyses (left and right lateral orbitofrontal cortex). Although there were no associations with cortical thickness in either the left or right lateral orbitofrontal cortex at corrected thresholds, higher average emotional granularity correlated with greater cortical thickness in both areas at a more relaxed threshold of *p<*.05, uncorrected. We controlled for age, sex, education, group (control or intervention), time interval (the number of days between the survey date and the MRI), and overall emotional experience intensity in these analyses. The color maps reflect the *T* values at *p<*.05, uncorrected.

**
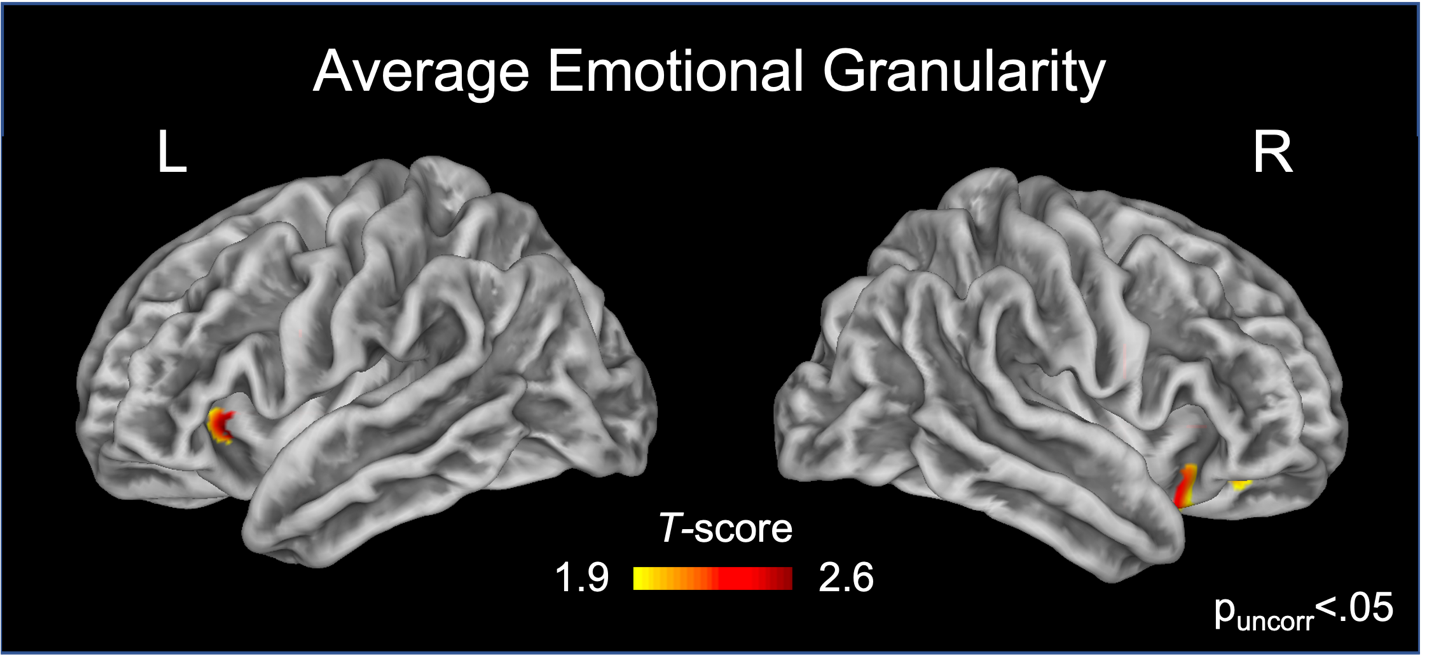
**

**Supplementary Table 1.** Demographic information and cognitive scores for the participants in the control and intervention groups in the study that was the source of the emotional experience data. Participants were randomly assigned to the control or intervention group and did not differ in any demographic or cognitive variables. Means (*M*) and standard deviations (*SD*) are provided.

|  | Control Group  *M (SD)* | Intervention Group *M (SD)* | *p* value |
| --- | --- | --- | --- |
| *N* | 30 | 28 |  |
| Age (years) | 73.8 (4.3) | 75.5 (4.2) | .152 |
| Sex (female / male) | 20 / 10 | 18 / 10 | 1.00 |
| Handedness (right / left) | 27 / 3 | 27 / 1 | .655 |
| Education (years) | 17.8 (2.0) | 17.1 (1.8) | .229 |
| Clinical Dementia Rating Scale Total | 0. 0 (0.0) | 0. 0 (0.0) | -- |
| Mini-Mental State Examination (/30) | 28.8 (1.4) | 29.6 (0.7) | .012 |
| California Verbal Learning Test-II Delayed Recall (/16) | 11.0 (3.5) | 12.0 (3.3) | .255 |
| Benson Figure Copy 10-minute Recall (/17) | 11.5 (2.4) | 11.2 (2.1) | .637 |
| Benson Figure Copy (/17) | 15.7 (0.6) | 15.4 (0.8) | .074 |
| Modified Trails (# of correct lines per minute) | 39.3 (12.4) | 41.6 (17.7) | .558 |
| Modified Trails Errors | 0.1 (0.6) | 0.3 (0.7) | .371 |
| Phonemic Fluency (# correct in 60s) | 15.8 (4.2) | 16.8 (4.6) | .379 |
| Semantic Fluency (# correct in 60s) | 21.9 (4.4) | 22.2 (4.3) | .854 |
| Design Fluency Correct (# correct in 60s) | 12.6 (3.9) | 11.7 (3.3) | .379 |
| Digits Backward | 5.3 (1.5) | 5.6 (1.2) | .340 |
| Boston Naming Test Spontaneous Correct (/15) | 14.6 (0.7) | 14.7 (0.6) | .644 |

**Supplementary Table 2.** Overall emotional granularity and emotional experience intensity scores are presented for the control and intervention groups that were the source of the emotional experience data. The groups did not differ in either measure. Means (*M*) and standard deviations (*SD*) are provided.

|  | Control Group  *M (SD)* | Intervention Group *M (SD)* | *p-value* |
| --- | --- | --- | --- |
| Overall Emotional Granularity (range: 0 – 1) | 0.90 (0.08) | 0.89 (0.06) | .711 |
| Overall Emotional Experience Intensity (/7) | 3.14 (0.95) | 3.32 (0.70) | .406 |
